# The endoscopic aspect of foraminal anatomy and dorsal root ganglion in percutaneous transforaminal endoscopic discectomy

**DOI:** 10.1002/ccr3.3347

**Published:** 2020-09-18

**Authors:** Stylianos Kapetanakis, Nikolaos Gkantsinikoudis, Tryfon Thomaidis, Michalis Georgoudis

**Affiliations:** ^1^ Spine Department and Deformities Interbalkan European Medical Center Thessaloniki Greece; ^2^ Department of Minimally Invasive and Endoscopic Spine Surgery Athens Medical Center Athens Greece

**Keywords:** neurosurgery, orthopaedics

## Abstract

Percutaneous Transforaminal Endoscopic Discectomy (PTED) offers an exceptional visualization of foraminal anatomy. Dorsal root ganglion and adjacent foraminal structures are satisfactorily visualized, thereby minimizing the risk of their intraoperative injury.

## Graphical abstract

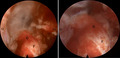

Percutaneous Transforaminal Endoscopic Discectomy (PTED) represents a novel minimally invasive method for surgical treatment of lumbar disc herniation (LDH). PTED provides access to the underlying pathology through Kambin's triangle, a right angle triangle located at the lower portion of lumbar intervertebral foramen (LIF). This triangle defined by the exiting nerve root as the hypotenuse, the posterolateral edge of superior endplate of underlying vertebra horizontally and the superior articular facet vertically.^1^ LIF constitutes a complex anatomic entity containing ligamentous, osseous, vascular, and neural elements as dorsal root ganglion (DRG). DRG represents the cluster of cell bodies of first‐order afferent sensory neurons. DRG features a proximal location to IVF in 11%‐38% and 71% of cases in L5 and S1 level, respectively.^2^ In our case, a 38‐year‐old man with right‐sided foraminal L4‐L5 LDH was successfully subjected to PTED. After excision of LDH with graspers (Figure 1) and identification of peri‐neural foraminal fat and facet joint synovial bursa (Figure 2), pulsatile nerve root as well as DRG and posterior radicular artery were surprisingly observed (Figures 3 and 4). Endoscopic emergence of DRG during PTED represents a potential scenario, and therefore, special care is required in order to avoid its intraoperative injury or irritation.

**FIGURE 1:**
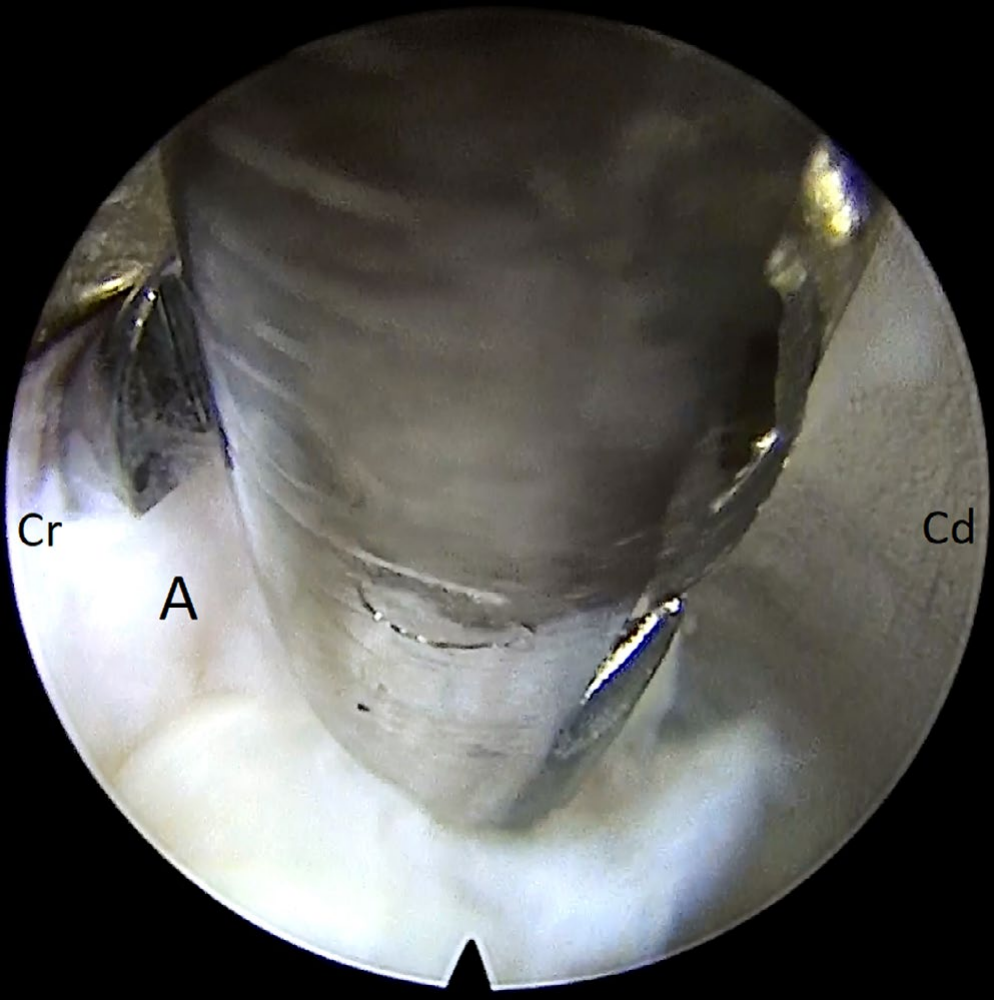
Remove of herniated nucleus pulposus (A) with graspers (Cr: cranial and Cd: caudal)

**FIGURE 2:**
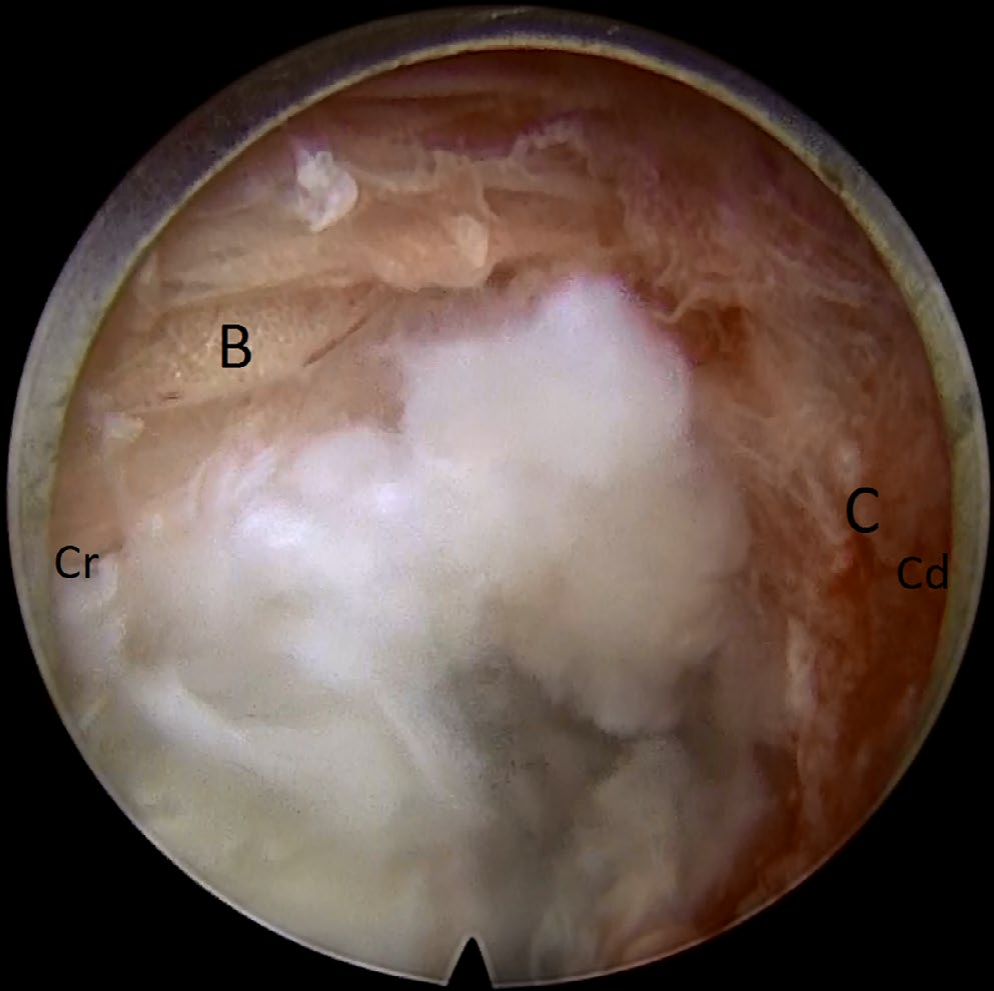
Identification of foraminal fat (B) surrounding the nerve root and the synovial bursa (C) of the facet joint (Cr: cranial and Cd: caudal)

**FIGURE 3:**
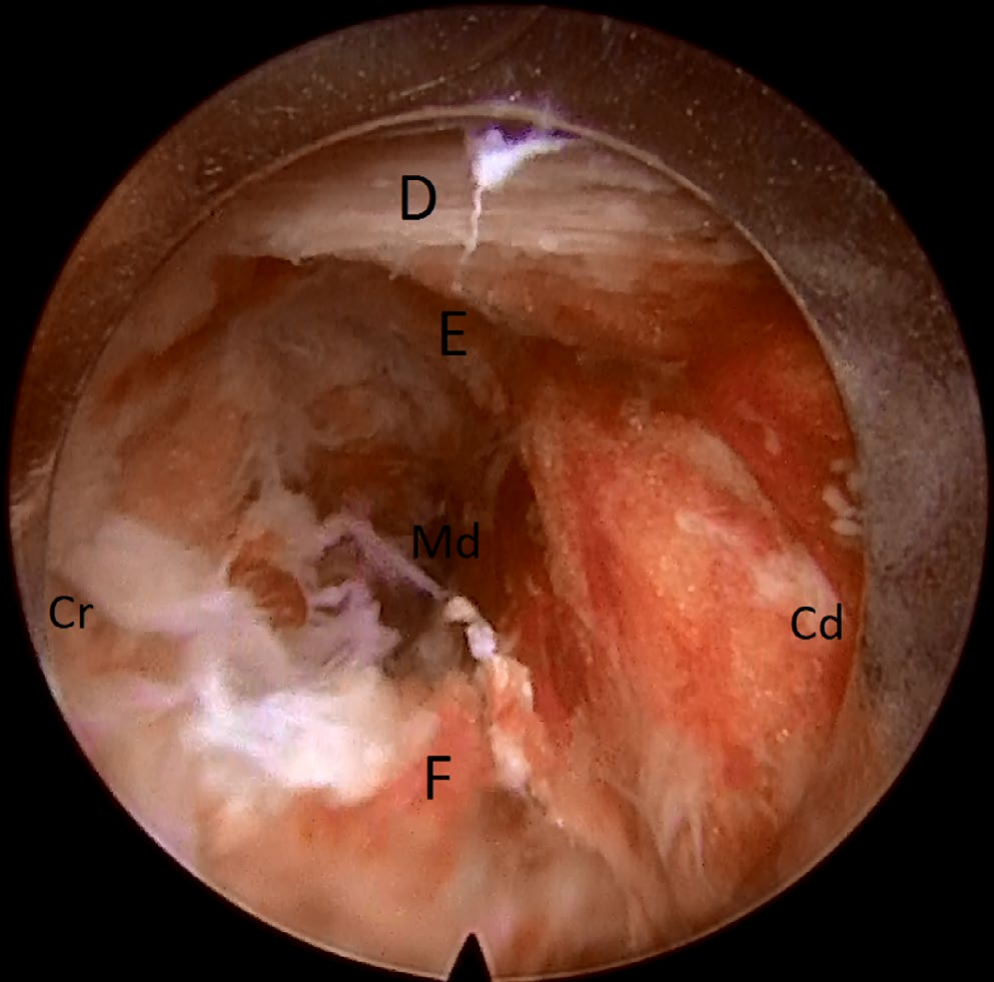
Subsequent identification of inferior corporotransverse ligament (D), pulsatile nerve root (E), and dorsal root ganglion (F) (Cr: cranial, Cd: caudal, and Md: medial)

**FIGURE 4:**
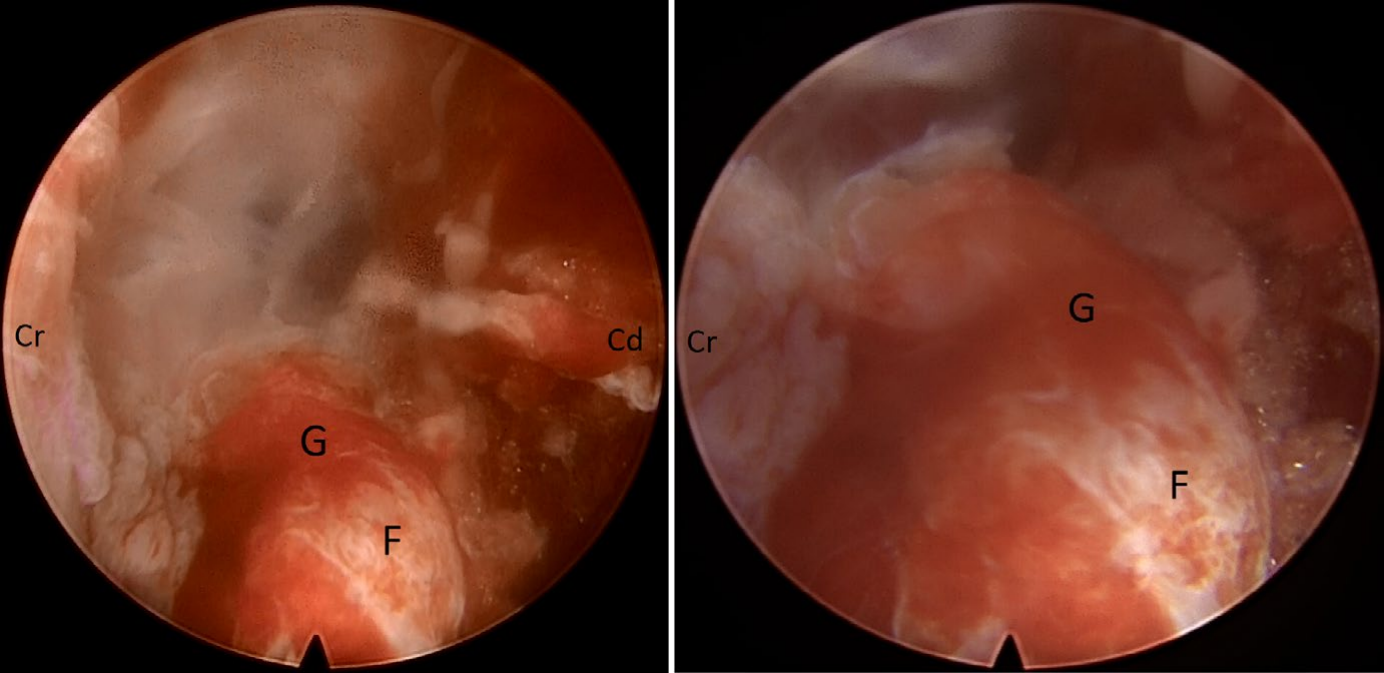
Endoscopic view of dorsal root ganglion (F) and respective posterior radicular artery (G) after meticulous nerve root decompression (Cr: cranial and Cd: caudal)

## CONFLICT OF INTEREST

Authors have no conflict of interest to declare.

## AUTHOR CONTRIBUTIONS

KS: involved in conception and design, acquisition of data, analysis and interpretation of data, critical revision of manuscript, general supervision, and final approval. GN: involved in acquisition of data, drafting of manuscript, and critical revision of manuscript. TT and GM: involved in acquisition of data, analysis and interpretation of data, and critical revision of manuscript.

## ETHICAL APPROVAL

Conduction and publication of this specific article was approved by Institutional Review Board and Ethics Committee of Interbalkan European Medical Center, Thessaloniki, Greece.
